# Diverse autoinhibitory mechanisms of FIIND-containing proteins: Insight into regulation of NLRP1 and CARD8 inflammasome

**DOI:** 10.1371/journal.ppat.1012877

**Published:** 2025-01-24

**Authors:** Jingfan Zhou, Chengrong Liu, Xin Wang, Zhenshan Liu, Zizhen Ming, Yonggang Wang, Chunxia Wang, Qiming Liang

**Affiliations:** 1 Institute of Pediatric Infection, Immunity, and Critical Care Medicine, Shanghai Children’s Hospital, Shanghai Jiao Tong University School of Medicine, Shanghai, China; 2 Shanghai Institute of Immunology, Department of Immunology and Microbiology, Key Laboratory of Cell Differentiation and Apoptosis of Chinese Ministry of Education, Shanghai Jiao Tong University School of Medicine, Shanghai, China; 3 Department of Cardiovascular Center, The First Hospital of Jilin University, Changchun, China; 4 Department of Critical Care Medicine, Shanghai Children’s Hospital, Shanghai Jiao Tong University School of Medicine, Shanghai, China; University of Southern California, UNITED STATES OF AMERICA

## Abstract

Function-to-find domain (FIIND)-containing proteins, including NLRP1 and CARD8, are vital components of the inflammasome signaling pathway, critical for the innate immune response. These proteins exist in various forms due to autoproteolysis within the FIIND domain, resulting in full-length (FL), cleaved N-terminal (NT), and cleaved C-terminal (CT) peptides, which form autoinhibitory complexes in the steady state. However, the detailed mechanism remains elusive. Here, we found that both NLRP1 paralogs and CARD8 form two conserved autoinhibitory complexes involving NT-CT interactions and FL-CT interactions, but with distinct mechanisms. Specifically, the Linker3 region located between LRR and FIIND in murine NLRP1b (mNLRP1b) plays an essential role in forming the NT-CT autoinhibitory complexes, while the ZU5 of rat NLRP1 (rNLRP1) and CARD8 mediates their NT-CT interaction. In addition, we explored the involvement of the cellular protease dipeptidyl peptidases 9 (DPP9) in these complexes, revealing differential interactions and the significance of domain structure. Besides the FL-DPP9-CT complex, DPP9 interacts with NTs of mNLRP1b, rNLRP1, and CARD8 through their ZU5 subdomains, forming NT-DPP9-CT complex; however, DPP9 cannot bind to NTs of hNLRP1. Further functional assay indicated that although DPP9 is involved in the NT-CT complex of rodent NLRP1 and CARD8, it does not influence the inhibitory activity of NT on CT. Our study enhanced the understanding of the regulatory functions of FIIND-containing proteins in inflammasome autoinhibition and activation and underscored the complexity of their interactions within the immune response.

## Author summary

Function-find domain (FIIND)-containing proteins, such as NLRP1 and CARD8, play a crucial role in the inflammasome signaling pathway, which is essential for the body’s innate immune response. These proteins undergo a process called autoproteolysis, resulting in different forms: full-length (FL), cleaved N-terminal (NT), and cleaved C-terminal (CT) peptides, which can form autoinhibitory complexes that help regulate immune signaling. In our research, we discovered that both NLRP1 paralogs and CARD8 form two main types of conserved autoinhibitory complexes involving interactions between NT and CT or between FL and CT. A specific region called Linker3 in murine NLRP1b is vital for its NT-CT interaction, while a different part known as ZU5 in rat NLRP1 and CARD8 mediates their NT-CT interactions as well. We also examined the role of a cellular protease, DPP9, finding that it forms complexes with NTs of rodent NLRP1 and CARD8, but does not affect their NT’s inhibitory functions. Our findings shed light on the complex regulatory mechanisms of FIIND-containing proteins in immune responses.

## Introduction

Inflammasomes are high-molecular-weight multiprotein complexes that play a critical role in detecting pathogenic microorganisms and endogenous danger signals, thereby mediating the activation of inflammatory caspases [1]. In canonical inflammasome pathways, nucleotide-binding-oligomerization-domain-like receptors (NLRs) sense pathogen-associated molecular patterns (PAMPs) or damage-associated molecular patterns (DAMPs), typically activating caspase-1 through the adaptor protein Apoptosis-associated speck-like protein containing a CARD (ASC) [1]. In noncanonical inflammasomes, murine caspase-11 or human caspase-4/5 directly recognizes bacterial lipopolysaccharide [2,3]. Activation of these caspases leads to the cleavage of gasdermin D (GSDMD), liberating the pore-forming GSDMD-N domain from the inhibitory GSDMD-C domain, resulting in pore formation in the plasma membrane and subsequent pyroptotic cell death [4,5]. Additionally, caspase-1 cleaves pro-IL-1β and pro-IL-18 to produce mature cytokines during canonical inflammasome activation; notably, human caspase-4 can directly process pro-IL-18 into mature IL-18 during noncanonical inflammasome activation [6].

Among the canonical inflammasome pattern recognition receptors (PRRs), both NLRP1 and CARD8 contain a unique function-to-find domain (FIIND), which undergoes autoproteolysis to yield two noncovalently associated polypeptides: the N terminus (NT) and the C terminus (CT) [7]. Human NLRP1 (hNLRP1) is structured with an N-terminal PYD, followed by the NACHT domain, LRR, FIIND, and a C-terminal CARD. Three linker regions separate these domains: Linker1 between PYD and NACHT, Linker2 between NACHT and LRR, and Linker3 between LRR and FIIND [8]. In contrast, murine NLRP1 paralogs lack the PYD and Linker1 regions [8]. Murine NLRP1b (mNLRP1b) recognizes *Bacillus anthracis* infection by detecting its bacterial protease, anthrax lethal toxin (LT). LT specifically cleaves the N-terminal fragment between amino acid 44 and 45, which triggers the proteasomal degradation of mNLRP1b^NT^ by the ubiquitin ligase UBR2, liberating mNLRP1b^CT^ for inflammasome assembly [9–11]. Additionally, the ubiquitin ligase IpaH7.8 from *Shigella Flexneri* also degrades mNLRP1b^NT^ via proteasomal degradation, activating the mNLRP1b inflammasome [10]. Unlike mNLRP1b, hNLRP1 is capable of sensing diverse stimuli during viral infection through various functional domains. For instance, the 3C protease of enterovirus cleaves hNLRP1 at Linker1, resulting in degradation of hNLRP1^NT^ and activation of the hNLRP1 inflammasome [12]. Semliki Forest virus activates the hNLRP1 inflammasome through viral dsRNA in keratinocytes, requiring the dsRNA-binding activity of LRR and the ATPase activity of NACHT [13]. Moreover, hNLRP1 directly interacts with the structural protein ORF45 during Kaposi’s sarcoma-associated herpesvirus (KSHV) infection via the Linker1 region, leading to the disruption of autoinhibitory complexes and activation of the hNLRP1 inflammasome [8]. Beyond viral infection, the hNLRP1 inflammasome also responds to ultraviolet B- and toxin-induced ribotoxic stress response in keratinocytes via cellular ZAKα kinase, which phosphorylates the Linker1 region of hNLRP1 [14,15]. Furthermore, dsDNA mimetic poly (dA:dT) and dsRNA mimetic poly (I:C) can trigger the hNLRP1 inflammasome in keratinocytes [13,16].

CARD8 shares similarities with NLRP1, possessing the FIIND-CARD regions but lacking the structural domains such as PYD, NACHT, and LRR. Instead, CARD8 contains a 160-amino-acid unstructured region in the N terminus [7]. The CARD8 inflammasome is activated by the protease activity during HIV-1 infection, which cleaves the N-terminal unstructured region [17]. The activation of the CARD8 inflammasome results in pyroptotic cell death in HIV-1-infected CD4^+^ T cells, further contributing to HIV-1 pathogenesis and disease progression [18]. Similarly, the viral protease of Coxsackie B3 virus cleaves the N-terminal region of CARD8, leading to inflammasome activation that is associated with viral myocarditis in cellular model [19].

The C-terminal CARD of NLRP1 and CARD8 is critical for inflammasome assembly. The NLRP1 inflammasome comprises NLRP1^CT^, ASC, and caspase-1, whereas the CARD8 inflammasome consists solely of CARD8^CT^ and caspase-1 [20,21]. The cellular DPP9 binds to the FIIND of NLRP1 or CARD8 and keeps them in an inactive state. The DPP9-FIIND complex, which includes DPP9, full-length NLRP1 or CARD8, and NLRP1^CT^ or CARD8^CT^, prevents activation of CT fragments without stimuli. Inhibition with the DPP8/9 inhibitor Val-boroPro (VbP) disrupts this inhibitory complex and allows for inflammasome activation through C-terminal fragment liberation [22–24]. Our recent study has also shown that hNLRP1 forms various autoinhibitory complexes with or without DPP9, indicating the Linker1 region plays a significant role in mediating the association between hNLRP1^NT^ and hNLRP1^CT^ [8]. Since Linker1 is unique to hNLRP1, the formation of similar autoinhibitory complexes with other NLRP1 paralogs or CARD8 is yet to be elucidated. It is believed that mNLRP1b^NT^ interacts with mNLRP1b^CT^ to silence the inflammasome activity [8–11]; however, the specifics of this interaction have not been clear demonstrated. Here, we utilized biochemistry approaches to characterize the autoinhibitory complexes of CARD8 and rodent NLRP1 variants, including mNLRP1b and rat NLRP1 (rNLRP1), revealing that all of these proteins form both NT-CT and FL-CT complexes. albeit through distinct mechanisms.

## Results

### FIIND-containing proteins form two distinct autoinhibitory complexes

**Fig 1:**
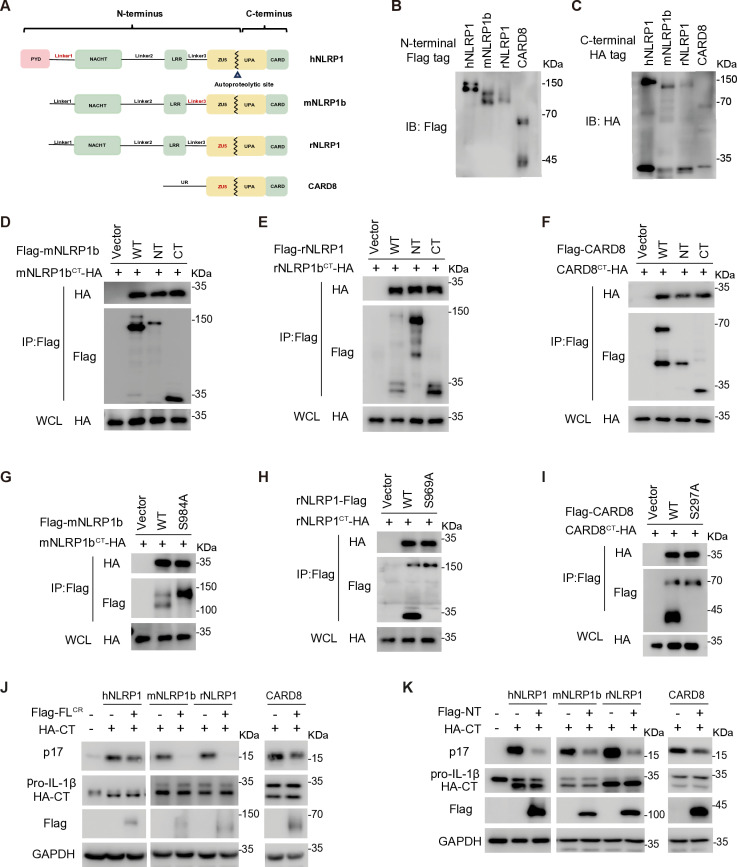
FIIND-containing proteins form two distinct autoinhibitory complexes. (**A**) Schematic diagram for the detailed domain arrangement for NLRP1 paralogs and CARD8. (**B and C**) Indicated plasmids with N-terminal Flag tag (B) or C-terminal HA tag (C) were transfected into HEK293T cells and cell lysates were subjected to immunoblot (IB) with indicated antibodies at 48 h post-transfection. (**D–F**) Interaction between CT and wild-type, NT, or CT of mNLRP1b (D), rNLRP1 (E), or CARD8 (F). HEK293T cell were transfected with indicated plasmids and cell lysates were subjected to immunoprecipitation (IP) and IB with indicated antibodies. WT, wild type. (**G–I**) Interaction between CT and wild-type or cleavage resistant mutant of mNLRP1b (G), rNLRP1 (H), or CARD8 (I). HEK293T cells were transfected with indicated plasmids and cell lysates were subjected to IP and IB with indicated antibodies. WT, wild type. (**J and K**) Cleavage resistant full length mNLRP1b, rNLRP1, or CARD8 (J) and NT of mNLRP1b, rNLRP1, or CARD8 (K) inhibit the corresponding CT inflammasome activity in the HEK293T reconstitution system. HEK293T cells were co-transfected with ASC, caspase-1, pro-IL-1β, and indicated FL^CR^, NT, or CT, and the cell lysates were subjected to IB with indicated antibodies at 36 h post-transfection. FL^CR^, cleavage resistant full length.

NLRP1 and CARD8 are the sole mammalian proteins containing FIIND domain and they exist in three forms at steady state: full-length protein (FL), cleaved N-terminal peptide (NT), and cleaved C-terminal peptide (CT). This diversity arises due to autoproteolysis occurring between ZU5 and UPA subdomains within FIIND (Fig 1A–1C). hNLRP1 forms two distinct autoinhibitory complexes in which both hNLRP1^FL^ and hNLRP1^NT^ interact with hNLRP1^CT^, effectively preventing the assembly of the inflammasome mediated by hNLRP1^CT^ [8]. The Linker1 region, located between PYD and NACHT of hNLRP1, is critical for forming these autoinhibitory complexes [8]; however, this region is not conserved in NLRP1 paralogs or CARD8. Consequently, we investigated whether other NLRP1 paralogs, such as mNLRP1b and rNLRP1, as well as CARD8, are capable of forming similar autoinhibitory complexes. In alignment with hNLRP1, co-immunoprecipitation assay conducted in HEK293T cells demonstrated that N-terminal Flag-tagged mNLRP1b^NT^, rNLRP1^NT^, and CARD8^NT^ can interact with their corresponding C-terminal HA-tagged CT counterparts (Fig 1D–1F). In addition to the NT-CT interaction, cleavage-resistant mutants—mNLRP1b^S984A^, rNLRP1^S969A^, and CARD8^S297A^ also interacted with their corresponding CT, consistent with previous reported interactions (Fig 1G–1I). These findings suggest that the formation of these two different complexes is a common feature shared by both CARD8 and NLRP1 paralogs.

Next, we assessed whether these complexes influenced the activation of the NLRP1 or CARD8 inflammasome. Both NLRP1^CT^ and CARD8^CT^ were capable of triggering the secretion of mature IL-1β in HEK293T cells expressing ASC, caspase-1, and pro-IL-1β (Fig 1J). In contrast, the expression of the corresponding cleavage-resistant forms of NLRP1 or CARD8 inhibited IL-1β maturation, preventing the activity of the corresponding CT (Fig 1J). Similarly, NLRP1^NT^ or CARD8^NT^ also exhibited an inhibitory effect on IL-1β secretion (Fig 1K). Given that CARD8 inflammasome activation does not depend on ASC [20,21], we focused on using ASC to evaluate NLRP1 inflammasome activation. All CTs from hNLRP1, mNLRP1b, and rNLRP1 induced the formation of ASC spikes in HEK293T cells stably expressing GFP-ASC, while expression of their corresponding cleavage-resistant NLRP1^FL^ or NLRP1^NT^ inhibited ASC spike formation (S1A–S1D Fig). These results demonstrate that both NLRP1 paralogs and CARD8 exist in a steady state characterized by the formation of NT-CT and FL-CT autoinhibitory complexes, suggesting a conserved regulatory mechanism among these FIIND-containing proteins.

### DPP9 is differently involved in the autoinhibitory complex of NLRP1 or CARD8

Previous structural studies have shown that the host protease DPP9 forms a ternary complex with FIIND^FL^ and UPA of hNLRP1, rNLRP1, and CARD8, effectively preventing UPA-mediated oligomerization and subsequent inflammasome activation [22–24]. However, these studies primarily focused on the FIIND domain or utilized proteins with significant deletions of their N-terminal domains [22–24]. As a result, it remains unclear whether other regions of NLRP1 or CARD8 contribute to DPP9 interaction or to the formation of autoinhibitory complex. Our prior research has demonstrated that NT-CT complex of hNLRP1 does not contain cellular DPP9 and the DPP8/9 inhibitor VbP only activates hNLRP1^FL^-DPP9-hNLRP1^CT^ complex, not hNLRP1^NT^-hNLRP1^CT^ complex [8]. This prompted us to investigate the role of DPP9 in the NT-CT and FL-CT complexes of mNLRP1b, rNLRP1, and CARD8.

**Fig 2:**
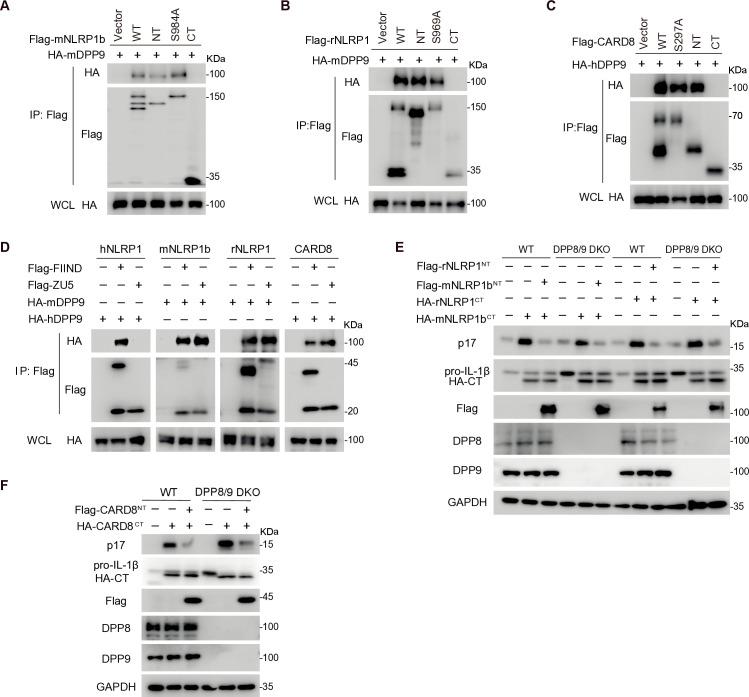
DPP9 is differently involved in the autoinhibitory complexes of NLRP1 or CARD8. (**A–C**) Interaction between DPP9 and NT or cleavage resistant mutants of mNLRP1b (A), rNLRP1 (B), and CARD8 (C). HEK293T cell were transfected with indicated plasmids and cell lysates were subjected to IP and IB with indicated antibodies. (**D**) Interaction between DPP9 and FIIND or ZU5 of NLRP1 paralogs and CARD8. HEK293T cells were transfected with indicated plasmids and cell lysates were subjected to IP and IB with indicated antibodies. (**E and F**) NTs of mNLRP1b, rNLRP1 (E) or CARD8 (F) inhibit their corresponding CT activity with or without DPP8/9 in the HEK293T reconstitution system. HEK293T or HEK293T^DPP8/9 DKO^ cells were co-transfected with ASC, caspase-1, pro-IL-1β, and indicated NT, or CT, and the cell lysates were subjected to IB with indicated antibodies at 36 h post-transfection.

In contrast with the behavior observed with hNLRP1, the cleavage resistant mutants of mNLRP1b (S984A), rNLRP1 (S969A), and CARD8 (S297A) showed similar binding affinity to DPP9 as the respective wild-type proteins, suggesting the autocleavage within the FIIND domain is not necessary for the interaction between DPP9 and rodent NLPR1s or CARD8 (Fig 2A–2C). In addition, the NT peptides of mNLRP1b, rNLRP1, or CARD8 also interacted with DPP9, whereas their CT peptides did not bind (Fig 2A–2C). Interestingly, a prior study indicated that DPP9 can also bind to the ZU5 subdomain of rNLRP1 independent of the FIIND-DPP9-UPA complex, suggesting a wider range of interactions [24]. Indeed, the ZU5 subdomain of rNLRP1, mNLRP1b, and CARD8 interacted with DPP9 as robustly as FIIND, while only the intact FIIND of hNLRP1 bound to DPP9 (Fig 2D). To assess whether DPP9 influences the formation of the mNLRP1b^NT^-mNLRP1b^CT^, rNLRP1^NT^-rNLRP1^CT^, or CARD8^NT^-CARD8^CT^ complexes, we generated a DPP8/9 double knockout HEK293T cell line using CRISPR-Cas9. In these knockout cells, the binding affinity between mNLRP1b^NT^ and mNLRP1b^CT^, between rNLRP1^NT^ and rNLRP1^CT^, or between CARD8^NT^-CARD8^CT^ remained unaffected (S2A–S2C Fig). These results indicate that while DPP9 interacts with mNLRP1b^NT^, rNLRP1b^NT^, and CARD8^NT^ through the ZU5 subdomain, it does not influence the formation of the respective NT-CT autoinhibitory complexes.

The DPP9 inhibitor VbP is known to activate hNLRP1, rNLRP1, mNLRP1b, and CARD8, largely due its ability to disrupt the FIIND-DPP9-UPA ternary complex (S2D Fig) [22–24]. Since DPP9 does not associate with hNLRP1^NT^ or hNLRP1^CT^, VbP only activates hNLRP1^FL^-DPP9-hNLRP1^CT^ complex but not hNLRP1^NT^-hNLRP1^CT^ complex [8]. We next determined whether DPP9 affects the activation of mNLRP1b^NT^-mNLRP1b^CT^, rNLRP1^NT^-rNLRP1^CT^, or CARD8^NT^-CARD8^CT^ complexes. We observed that much like hNLRP1, rNLRP1^NT^, mNLRP1b^NT^, and CARD8^NT^ continued to inhibit the activation of their corresponding CTs, regardless of DPP8/9 knockout (Fig 2E and 2F). Additionally, VbP did not enhance the activation of the NT-CT complexes of mNLRP1b, rNLRP1, or CARD8 (S2E Fig). These findings indicate that although DPP9 can interact with mNLRP1b^NT^-mNLRP1b^CT^, rNLRP1^NT^-rNLRP1^CT^, and CARD8^NT^-CARD8^CT^ complexes, it does not have an effect on the inhibitory activity of the NTs over their respective CTs.

### Characterization of the NT-CT autoinhibitory complex of mouse NLRP1b

**Fig 3:**
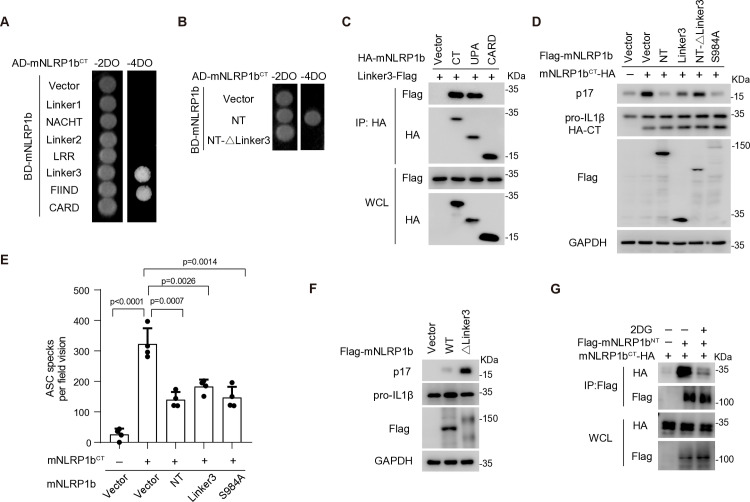
Characterization of the NT-CT autoinhibitory complex of mouse NLRP1b. (**A and B**) Interaction assay between mNLRP1b^CT^ and each individual domain of mNLRP1b (A) or mNLRP1b mutant (B) by the yeast two-hybrid assay. (**C**) Interaction between Linker3 and UPA in HEK293T cells. HEK293T cell were transfected with indicated plasmids and cell lysates were subjected to IP and IB with indicated antibodies. (**D**) Linker3 blocks mNLRP1b^CT^-mediated inflammasome activation in the HEK293T reconstitution system. HEK293T cells were co-transfected with ASC, caspase-1, pro-IL-1β, mNLRP1b^CT^, and indicated mutants of mNLRP1b, and the cell lysates were subjected to IB with indicated antibodies at 36 h post-transfection. (**E**) Quantification of ASC spike microscopy images in GFP–ASC HEK293T cells transfected with the indicated expression vectors for 36 h (n = 4). (**F**) Mature IL-1β (p17) generation in ASC–caspase-1–pro-IL-1β HEK293T cells transfected with wild-type mNLRP1b or mNLRP1b^ΔLinker3^. (**G**) 2DG blocks mNLRP1b^NT^-mNLRP1b^CT^ interaction. HEK293T cells were co-transfected with indicated plasmids, followed by 2DG (50 mM) treatment for 6 h. Cell lysates were subjected to IP and IB with indicated antibodies at 48 h post-transfection. All data represent three independent experiments and are presented as mean ± s.d. For statistical analysis, two-tailed unpaired Student’s *t*-test in (E).

We previously demonstrated that the Linker1 region between the PYD and NACHT domains mediates the formation of the hNLRP1^NT^-hNLRP1^CT^ complex through an association with the UPA subdomain [8]. Although mNLRP1b lacks the N-terminal PYD and Linker1 region, mNLRP1b^NT^ still interacts with mNLRP1b^CT^ (Fig 1D). To identify the interaction regions of mNLRP1b that facilitate this binding, we employed a yeast two-hybrid system. Similar to hNLRP1, the FIIND domain of mNLRP1b was found to interact with mNLRP1b^CT^, which supports the formation of the FIIND-DPP9-UPA ternary complex [22,24], while the Linker3 region located between LRR and FIIND showed similar binding affinity to mNLRP1b^CT^ (Fig 3A). Additionally, the Linker3 region also exhibited similar binding affinity to mNLRP1b^CT^ in cellular assay (S3A Fig), and deletion of Linker3 abolished the association between mNLRP1b^NT^ and mNLRP1b^CT^ (Fig 3B). Further mapping indicated that the UPA subdomain, but not the CARD domain of mNLRP1b^CT^, was associated with Linker3 (Fig 3C). Within the FIIND domain, only the UPA but not ZU5 subdomain was responsible for the interaction with mNLRP1b^CT^ (S3B Fig), supporting the idea that UPA-mediated oligomerization is a common mechanism for NLRP1 inflammasome activation [20,21]. These results demonstrate that the association between Linker3 and UPA is critical for the interaction between mNLRP1b^NT^ and mNLRP1b^CT^.

Linker3 comprises 145 amino acids located between the LRR and FIIND domains of mNLRP1b (S3C Fig). Detailed mapping revealed that aa 85–145 of Linker3 (corresponding to aa 789–849 in full-length mNLRP1b) was responsible for the association with mNLRP1b^CT^, while the first 84 amino acids of Linker3 (corresponding to aa 705–788 in full length mNLRP1b) did not contribute to this interaction (S3D Fig). Notably, aa 85–145 of Linker3 is unique to mNLRP1b allele1 and are absent in allele 2–5, which arose from the duplication of two exons during evolution (S3C Fig) [25,26]. This suggests that the Linker3-mediated mNLRP1b^NT^-mNLRP1b^CT^ complex is specific to allele1 of mNLRP1b.

Next, we investigated the role of Linker3 in the autoinhibition of the mNLRP1b inflammasome. Similar to hNLRP1^CT^, individual expression of mNLRP1b^CT^ induced IL-1β production in HEK293T cells expressing ASC, caspase-1, and pro-IL-1β (Fig 3D). Co-expression of the Linker3 region was sufficient to inhibit mNLRP1b^CT^-mediated inflammasome activation, whereas deletion of Linker3 eliminated the inhibitory activity of mNLRP1b^NT^ on mNLRP1b^CT^ (Fig 3D). Consistently, Linker3 also blocked the mNLRP1b^CT^-mediated ASC spike formation in HEK293T cells stably expressing GFP-ASC, similar to the effect of mNLRP1b^NT^ or the cleavage-resistant mNLRP1b^S984A^ (Fig 3E). Because of the critical role of Linker3 in the formation of the mNLRP1b^NT^-mNLRP1b^CT^ complex, deletion of Linker3 from full-length mNLRP1b resulted in spontaneous activation of the mNLRP1b inflammasome (Figs 3F and S3E). These data establish that the Linker3 region is both necessary and sufficient for the formation of the mNLRP1b^NT^-mNLRP1b^CT^ autoinhibitory complex.

Previous reports have indicated that LF activates the mNLRP1b inflammasome through a mechanism termed “functional degradation” [9–11]. In the mNLRP1b^NT^-mNLRP1b^CT^ autoinhibitory complex, LF induces degradation of mNLRP1b^NT^, leading to activation of mNLRP1b^CT^-mediated inflammasome, suggesting that LF may activate the mNLRP1^NT^-mNLRP1^CT^ autoinhibitory complex (S3F Fig). Additionally, the mNLRP1b inflammasome is known to sense metabolic stress during conditions of glucose deprivation or upon treatment with metabolic inhibitor [26,27]. For example, 2-Deoxy-D-glucose (2DG), a glycolysis inhibitor, triggers pyroptotic cell death that can be prevented by deletion of mNLRP1b [26,27]. Furthermore, deletion of the duplication of two exons in Linker3 region of mNLRP1b removes its capacity to respond to 2DG treatment [26,27], suggesting that 2DG may target the mNLRP1b^NT^-mNLRP1b^CT^ autoinhibitory complex. In line with this, we observed that 2DG disrupted the interaction between mNLRP1b^NT^ and mNLRP1b^CT^, thereby diminishing the inhibitory effect of mNLRP1b^NT^ on mNLRP1b^CT^ activation (Figs 3G and S3G). These findings indicate that mNLRP1b^NT^ is capable of sensing LF or 2DG and the Linker3 region plays a critical role in the formation of the mNLRP1b^NT^-mNLRP1b^CT^ autoinhibitory complex.

### Characterization of the NT-CT autoinhibitory complex of rat NLRP1

**Fig 4:**
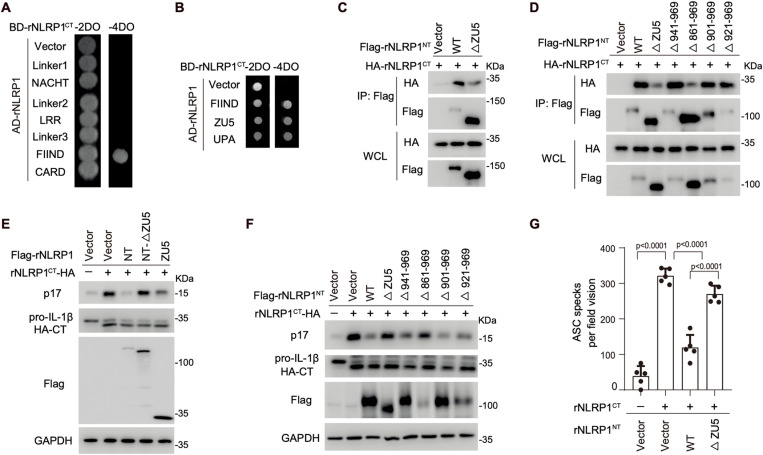
Characterization of the NT-CT autoinhibitory complex of rat NLRP1. (**A and B**) Interaction assay between rNLRP1^CT^ and each individual domain (A) or subdomains (B) of rNLRP1 by the yeast two-hybrid assay. (**C**) ZU5 is required for rNLRP1^NT^-rNLRP1^CT^ interaction. HEK293T cell were transfected with indicated plasmids and cell lysates were subjected to IP and IB with indicated antibodies. (**D**) Mapping the critical region within ZU5 for rNLRP1^NT^-rNLRP1^CT^ interaction. (**E**) ZU5 is necessary and sufficient to inhibit rNLRP1^CT^ activity. HEK293T cells were co-transfected with ASC, caspase-1, pro-IL-1β, and indicated rNLRP1 fragments or mutants, and the cell lysates were subjected to IB with indicated antibodies at 36 h post-transfection. (**F**) Mapping the critical region within ZU5 for rNLRP1^CT^ inhibition. (**G**) ASC spike quantification in GFP-ASC HEK293T cells transfected with indicated plasmids for 36 h (n = 3). All data represent three independent experiments and are presented as mean ± s.d. For statistical analysis, two-tailed unpaired Student’s *t*-test in (G).

NLRP1 exhibits significant polymorphism among inbred rodent strains, with mouse strains expressing three paralogs: mNLRP1a, mNLRP1b, and mNLRP1c. In contrast, rats possess a single NLRP1 gene that is polymorphic across different alleles [25]. Notably, rNLRP1 lacks the N-terminal PYD and Linker1 but maintains a similar domain arrangement to mNLRP1b. To determine whether rNLRP1 employs a similar strategy to form autoinhibitory complexes as seen in mNLRP1b, we conducted yeast two-hybrid mapping. We found that FIIND domain of rNLRP1 bound to rNLRP1^CT^, akin to the interaction observed in mNLRP1b (Fig 4A). However, unlike mNLRP1b, the Linker3 region did not interact with rNLRP1^CT^ (Fig 4A), suggesting that rNLRP1 utilizes different mechanisms for autoinhibitory complex formation. Further mapping within the FIIND domain revealed that both the UPA and ZU5 subdomains can interact with rNLRP1^CT^ (Figs 4B and S4A). Additionally, the ZU5 subdomain specifically associate with the UPA, but not the CARD domain, of rNLRP1^CT^ ( Fig S4B). Importantly, deletion of the ZU5 subdomain significantly reduced the interaction between rNLRP1^NT^ and rNLRP1^CT^ in HEK293T cells (Fig 4C), indicating that ZU5 is essential for the formation of the rNLRP1^NT^-rNLRP1^CT^ complex.

The ZU5 subdomain can bind to rNLRP1^CT^ in both DPP9-dependent and DPP9-independent manners, participating in the formation of both the FL-CT complex and the NT-CT complex. This is consistent with the findings from cryo-EM structural studies of the rNLRP1 auto-inhibitory complex. The ZU5 subdomain comprises 135 aa (corresponding to aa 835–969 of rNLRP1) (S4C Fig). To further characterize how ZU5 interacts with rNLRP1^CT^, we generated a series of C-terminal truncated mutants of rNLRP1^NT^ and evaluated their binding to rNLRP1^CT^. Mutants rNLRP1^NT-Δ943–969^, rNLRP1^NT-Δ921–969^, and rNLRP1^NT-Δ901–969^ retained the ability to bind rNLRP1^CT^, while rNLRP1^NT-Δ861–969^ lost the capability (Figs 4D and S4C). This suggests that aa 861–900 within ZU5 is involved in the formation of the rNLRP1^NT^-rNLRP1^CT^ complex.

Next, we assessed the role of ZU5 in modulating rNLRP1^CT^-mediated inflammasome activation. Individual expression of rNLRP1^CT^ triggered IL-1β production in HEK293T cells expressing ASC, caspase-1, and pro-IL-1β (Fig 4E). However, co-expression of ZU5 blocked rNLRP1^CT^ activation, similar to the effect of rNLRP1^NT^ (Fig 4E). Furthermore, deletion of ZU5 from rNLRP1^NT^ (rNLRP1^NT-ΔZU5^) abolished its inhibitory effect on rNLRP1^CT^ activation (Fig 4E), indicating that ZU5 is both necessary and sufficient to mediate the formation of the rNLRP1^NT^-rNLRP1^CT^ autoinhibitory complex. In agreement with the binding results, rNLRP1^NT-Δ861–969^ cannot inhibit rNLRP1^CT^ activation, whereas mutants rNLRP1^NT-Δ943–969^, rNLRP1^NT-Δ921–969^, and rNLRP1^NT-Δ901–969^ exhibited similar inhibitory effect as rNLRP1^NT^ (Fig 4F). Consistently, rNLRP1^NT^ and the ZU5 subdomain inhibited rNLRP1^CT^-mediated ASC spike formation, while the rNLRP1^NT-Δ861–969^ mutant failed to do so (Figs 4G and S4D). These findings confirm that the ZU5 subdomain plays a critical role in the formation of the rNLRP1^NT^-rNLRP1^CT^ autoinhibitory complex.

Previous reports indicated that certain inbred rat strains and their macrophages are sensitive to LF challenge [28,29]. Similar to mNLRP1b, LF induced the degradation of rNLRP1^NT^, facilitating the release of bioactive rNLRP1^CT^ from its auto-inhibitory complexes (S4E Fig). These results demonstrate that LF can activate rNLRP1 by disrupting the rNLRP1^NT^-rNLRP1^CT^ complex.

### Characterization of the NT-CT autoinhibitory complex of CARD8

**Fig 5:**
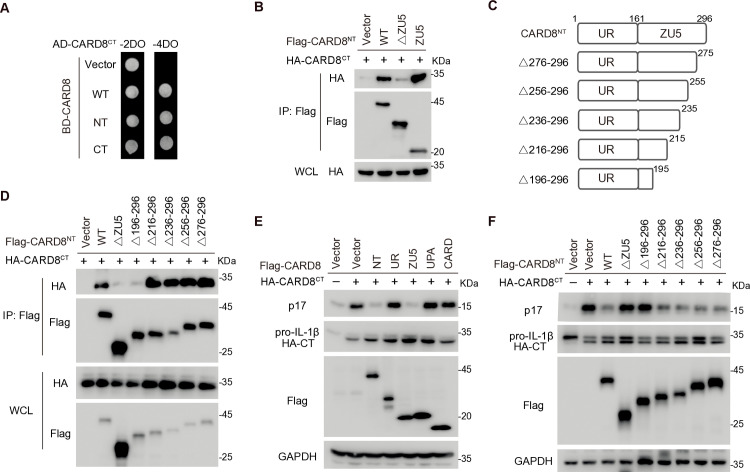
Characterization of the NT-CT autoinhibitory complex of CARD8. (**A**) Interaction assay between CARD8^CT^ and other domains by the yeast two-hybrid assay. (**B**) ZU5 is required for rNLRP1^NT^-rNLRP1^CT^ interaction. HEK293T cell were transfected with indicated plasmids and cell lysates were subjected to IP and IB with indicated antibodies. (**C**) Domain architecture of CARD8. (**D**) Mapping the critical region within ZU5 for CARD8^NT^-CARD8^CT^ interaction. (**E**) ZU5 blocks CARD8^CT^ activity. HEK293T cells were co-transfected with ASC, caspase-1, pro-IL-1β, and indicated CARD8 fragments or mutants, and the cell lysates were subjected to IB with indicated antibodies at 36 h post-transfection. (**F**) Mapping the critical region within ZU5 for CARD8^NT^-mediated CARD8^CT^ inhibition. Mapping the critical region within ZU5 for CARD8^NT^-CARD8^CT^ interaction.

Aside from NLRP1, CARD8 is the only other protein containing FIIND domain that undergoes post-translational autoproteolysis. While CARD8 and NLRP1 share similar C-terminal regions, CARD8 lacks a structured N-terminal region, possessing only a 160-aa N-terminal unstructured region (UR). Despite this minimal structure, CARD8^NT^ is capable of interacting with CARD8^CT^ in both HEK293T cells and yeast two-hybrid system (Figs 1F and 5A). The interaction between CARD8^NT^ and CARD8^CT^ was mediated by the ZU5 subdomain, rather than the unstructured N-terminal region (Fig 5B), indicating that CARD8 employs a mechanism akin to rNLRP1 for the formation of the CARD8^NT^-CARD8^CT^ complex. To further delineate the specific regions within ZU5 that are necessary for CARD8^NT^-CARD8^CT^ interaction, we generated a series of C-terminal truncated mutants of CARD8^NT^ and evaluated their interaction with CARD8^CT^ (Fig 5C). Mutants such as CARD8^NT-Δ216–296^, CARD8^NT-Δ236–296^, CARD8^NT-Δ256–296^, and CARD8^NT-Δ276–296^ demonstrated comparable binding affinity to CARD8^CT^ as the wild-type CARD8^NT^, while mutants like CARD8^NT-Δ196–296^ and CARD8^NT-Δ161–296^ (ΔZU5) failed to interact with CARD8^CT^. These results pinpoint aa 196–215 are critical for the interaction between CARD8^NT^ and CARD8^CT^ (Fig 5D).

Next, we examined the role of ZU5 in modulating CARD8^CT^-driven inflammasome activation. CARD8^CT^ directly interacts with caspase1 in an ASC-independent manner [20,21], and individual expression CARD8^CT^ induced IL-1β production in HEK293T cells expressing caspase-1 and pro-IL-1β (Fig 5E). Co-expression of either CARD8^NT^ or ZU5 effectively inhibited CARD8^CT^-mediated inflammasome activation, whereas the deletion of ZU5 from CARD8^NT^ (CARD8^UR^) nullified this inhibitory effect (Fig 5E). This indicates that ZU5 is both necessary and sufficient for suppressing CARD8^CT^ activation within the CARD8^NT^-CARD8^CT^ auto-inhibitory complex. Consistent with the binding data, CARD8^NT-Δ196–296^ mutant could not inhibit CARD8^CT^ activity, similar to CARD8^NT-ΔZU5^, while mutants CARD8^NT-Δ216–296^, CARD8^NT-Δ236–296^, CARD8^NT-Δ256–296^, and CARD8^NT-Δ276–296^ retained the ability to suppress CARD8^CT^-mediated inflammasome activation (Fig 5F). These results underscore the critical role of aa 196–215 within ZU5 for the formation of the CARD8^NT^-CARD8^CT^ autoinhibitory complex.

## Discussion

The formation of distinct autoinhibitory complexes involving FIIND-containing proteins, such as NLRP1 and CARD8, represents a highly regulated mechanism in controlling inflammasome activation. Our findings demonstrate that both NLRP1 paralogs and CARD8 form two conserved autoinhibitory complexes, including NT-CT and FL-CT interactions, but with distinct molecular mechanisms. Both the NT and FL proteins bind to CT and restrict CT’s activity for inflammasome assembly and activation. The FIIND domain of FL proteins forms a relatively conserved ternary complex with the cleaved CT and the cellular protease DPP9 [22–24], while NT utilizes different domains or regions to interact with and inhibit CT (S4F Fig). Specifically, hNLRP1^NT^ employs its Linker1 region (between PYD and NACHT domains) to bind to hNLRP1^CT^ [8], whereas mNLRP1b^NT^ utilizes Linker3 (between LRR and FIIND) to interact with mNLRP1b^CT^. Both rNLRP1^NT^ and CARD8^NT^ exploit the ZU5 subdomain for their corresponding CT interactions. The UPA subdomain in the CTs mediates interactions with Linker1 (hNLRP1^NT^), Linker3 (mNLRP1b^NT^), or ZU5 (rNLRP1^NT^ and CARD8^NT^), involving in the inflammasome assembly through self-oligomerization.

Consequently, the deletion of Linker1 from hNLRP1 or Linker3 from mNLRP1b not only abolishes NT-CT interaction but also results in spontaneous inflammasome activation. Although NLRP1 homologs exhibit low sequence identity in the N-terminus between humans and rodents, they all can form NT-CT autoinhibitory complexes. Notably, mNLRP1b demonstrates high polymorphism in mice, with five different allelic variants. Only the allele1 possesses the NLRP1b^NT^-NLRP1^CT^ complex, attributed to a 61-amino-acid insertion in Linker3, precisely at the binding site for mNLRP1b^CT^. This could help explain why mNLRP1b from C57BL/6J mice (allele2) exhibits higher basal inflammasome activity compared to mNLRP1b from Balb/C mice (allele1) [30]. These findings suggest that similar autoinhibitory mechanisms abide across diverse species for FIIND-containing proteins, indicating an evolutionarily conserved strategy to regulate inflammasome signaling.

Cellular DPP9 has been identified as a critical checkpoint for both NLRP1 and CARD8 inflammasomes through the formation of similar complexes (FL-DPP9-CT), dependent on autoproteolysis within the FIIND domain. Indeed, DPP9 binds only to full-length hNLRP1 but not its NTs or CTs; mutations at the cleavage sites of hNLRP1 (S1213A) eliminate their interactions with DPP9. However, mNLRP1b,rNLRP1, and CARD8 present a different profile, as both FL and NT can bind DPP9 with similar affinity, and mutations at the cleavage sites do not impact this interaction. Detailed mapping further indicates that the ZU5 subdomain of mNLRP1b, rNLRP1 and CARD8 also binds to DPP9, suggesting that DPP9 is involved in both FL-CT and NT-CT complexes. DPP9 restricts CT’s activity to prevent inflammasome activation in the FL-DPP9-CT complex, whereas it does not affect CT’s function or NT’s inhibitory activities in the NT-DPP9-CT complex for mNLRP1b, rNLRP1, and CARD8. Knockout studies indicate that NT retains its inhibitory effect on CT in the absence of DPP9, and treatment with VbP does not activate the NT-DPP9-CT complex, suggesting that the primary inhibitory action of DPP9 for NLRP1 or CARD8 occurs through the FL-DPP9-CT complex.

Both NLRP1 paralogs and the CARD8 inflammasome can be activated by pathogenic proteases through a mechanism termed “functional degradation”, whereby these proteases cleave the NT, leading to the degradation of both NT and FL, thus liberating CT for inflammasome assembly [9–12,17,19]. Consequently, these viral or bacterial proteases can activate both NT-CT and FL-CT autoinhibitory complexes. Furthermore, the disruption of the interaction between NT and CT provides an additional activation mechanism for the NLRP1 inflammasome. For instance, KSHV ORF45 binds to the Linker1 region of hNLRP1 and inhibits the association between hNLRP1^NT^ and hNLRP1^CT^ to activate the hNLRP1 inflammasome [8]. However, KSHV ORF45 cannot activate rodent NLRP1 inflammasome since rodent NLRP1 paralogs do not have Linker1. Similarly, 2DG, a glycolysis inhibitor, prevents the mNLRP1b^NT^-mNLRP1b^CT^ association, thereby activating the mNLRP1b inflammasome [26,27]. Future studies examining the interplay between metabolic states and inflammasome activation will be essential for understanding how these pathways converge in health and disease.

In conclusion, our research elucidates that the formation of NT-CT autoinhibitory complexes in FIIND-containing proteins constitutes a sophisticated mechanism of regulation, presenting potential avenues for targeted therapeutic intervention in diseases associated with dysregulated inflammasome activation. Further investigation into the structure-function relationships within these complexes will enhance the understanding of their biological significance and therapeutic potential.

## Methods

### Cells

HEK293T cells were maintained in DMEM supplemented with 10% FBS, 2 mM L-glutamine, 100 units/ml penicillin and 100 mg/ml streptomycin at 37 °C in a 5% CO2 incubator. For generation of knockout cells by the CRISPR–Cas9 system, lentiviruses expressing Cas9 and the desired sgRNA with a ZsGreen selection marker were generated and used to infect HEK293T cells. ZsGreen-positive cells were sorted by flow cytometry (BD Biosciences FACS Aria II) and validated for specific gene knockout by immunoblotting with specific antibodies at 48 h after infection. Sequences of the sgRNA species are 5ʹ-TACGTGATAAATTCCACTAC-3ʹ for DPP8, 5ʹ-GGCCAACATCGAGACAGGCG-3ʹ for DPP9 and 5ʹ-ACGGAGGCTAAGCGTCGCAA-3ʹ for the non-targeting control.

### Plasmid constructs

cDNA encoding hNLRP1, ASC, caspase-1 was provided by the Core Facility of Basic Medical Sciences, Shanghai Jiao Tong University School of Medicine. Murine NLRP1b, Rat NLRP1 and CARD8 were synthesized by GenScript Biotech. For yeast two-hybrid analysis, genes encoding rodent NLRP1 or CARD8 and their mutants were subcloned into pGBKT7 (BD, DNA-binding domain) or pACT2 (AD, transcript activated domain) vectors as indicated. For cellular expression, the indicated genes and their truncated mutants, point mutation or internal deletion mutants were cloned into the pEF vector or the pKH3 vector. The Flag tag was fused to the N-terminus of FL or NT for both NLRP1 paralogs and CARD8. The HA tag was fused to the C-terminus of CT for both NLRP1 paralogs and CARD8. For generating stable cell lines, DNA coding for ASC was cloned into the pLVX vector with the sequence for the GFP tag. The point mutations or internal deletions were generated using the ClonExpress II One Step Cloning kit (Vazyme Biotech, C122-01). All constructs were sequenced using an ABI PRISM 377 automatic DNA sequencer to verify 100% correspondence with the original sequence.

### Immunoprecipitation and immunoblotting

For co-immunoprecipitation, 2 × 10^6^ HEK293T cells were transfected with 20 μg of plasmid at a confluency of 90% with Lipofectamine 3000 (Thermo Fisher Scientific, #3000015). The cells were washed twice with cold phosphate-buffered saline (PBS) and lysed in a whole cell lysis buffer (WCL) containing (50 mM Tris·HCl [pH 7.4], 150 mM NaCl, 1% NP-40, 1 mM EDTA, 10% glycerol, protease inhibitor cocktail [Roche]) for 20 min on ice at 48 h post-transfection. The cell lysates were then centrifuged at 15,000 g for 15 min and the clear supernatants were subjected to immunoprecipitation with anti-Flag M2 agarose resin (Sigma-Aldrich, #F2426) following the manufacturer’s instruction. After 4 h incubation at 4 °C, the beads were washed for three times with WCL and twice with PBS, and then boiled with the 2×loading buffer for 10 min. The immunoprecipitants were applied to standard immunoblotting analyses with indicated specific antibodies. Primary antibodies used in this study include: rabbit monoclonal anti-cleaved-IL-1β (p17) (CST, #83186; 1:2000; clone D3A3Z), rabbit polyclonal anti-DPP8 (Abcam, #ab96470; 1:1000), mouse monoclonal anti-DPP9 (Santa Cruz, #sc-271634; 1:1000; clone F-1), mouse monoclonal anti-Flag, HRP conjugated (BioLegend, #637311; 1:5000; clone L5), mouse monoclonal anti-HA, HRP conjugated (BioLegend, #901519; 1:5000; clone 16B12).

### Recombined NLRP1 inflammasome activation

HEK293T cells were seeded into a 12-well plate (4 × 10^5^ cells per well) and incubated overnight. pEF-Casp1-V5 (10 ng), pEF-ASC-V5 (10 ng) and pEF-pro-IL-1β-HA (400 ng) were co-transfected with other plasmids on the 2nd day. Cells were collected and subjected to immunoblotting for mature IL-1β (p17) at 36 h after transfection.

### ASC speck-formation assay

GFP–ASC HEK293T single clones with low background of ASC aggregation without any stimulation were selected. Cells were seeded into 6-well plates (4 × 10^5^ cells per well), incubated overnight and then transiently transfected with 100 ng of the indicated plasmids on the 2nd day. Transfected cells were imaged by fluorescence microscopy at 36 h after transfection. For each group, several randomly selected fields with similar cell confluency were analyzed. ASC specks were quantified using ImageJ software. Similar results were obtained in at least three independent experiments.

### Yeast two-hybrid analysis

The yeast two-hybrid screen was performed as described previously. Briefly, the Y2HGold Yeast Strain (Clontech, #630498) that contains four integrated reporter genes under the control of three distinct Gal4-responsive promoters was used to detect two-hybrid interactions. Different combinations of the pGBKT7 (DNA-binding domain) and pACT2 (activation domain) constructs were simultaneously transformed using the Yeast maker Yeast Transformation System 2 (Clontech, #63039), following the manufacturer’s manual. Transformed yeast cells were washed once with sterile water and resuspended in sterile water and spotted onto −2DO plates (SD, −Leu, −Trp dropout medium) to assess transformation efficiency and onto −4DO plates (SD, −Ade, −His, −Leu, −Trp selection medium) to evaluate the potential interactions. SD, minimal synthetic defined bases. Plates were incubated for 4–6 d at 30 °C for positive clone growth. All constructs were tested for auto-activating properties to confirm the lack of autonomous activation on the reporter genes. All positive interactions were confirmed with at least three technical replicates.

### Quantification and statistical analysis

All data were expressed as Mean ± s.d., unless otherwise noted. For parametric analysis, the F test was used to determine the equality of variances between the groups compared; statistical significance across two groups was tested by two-tailed unpaired Student’s t-test; one-way analysis of variance (ANOVA) followed by Bonferroni’s *post hoc* test were used to determine statistically significant differences between multiple groups. *P*-values of less than 0.05 were considered significant.

## Supporting information

S1 Fig FIIND-containing proteins form two distinct autoinhibitory complexes. (**A and B**) ASC spike microscopy images (A) and quantification (B) of GFP–ASC HEK293T cells transfected with expression vectors encoding NLRP1^CT^ and NLRP1^NT^ from different species as indicated for 36 h (n = 5). Scale bar, 50 μm. (**C and D**) ASC spike microscopy images (C) and quantification (D) of GFP–ASC HEK293T cells transfected with expression vectors encoding NLRP1^CT^ and NLRP1 cleavage-resistant (CR) mutants from different species as indicated for 36 h (n = 5). Scale bar, 50 μm. All data represent three independent experiments. For statistical analysis, two-tailed paired Student’s *t*-tests in (B, D).
(TIF)

S2 Fig DPP9 does not affect the NT-CT autoinhibitory complexes of NLRP1 or CARD8. (**A–C**) Interaction between NT and CT of mNLRP1b (A), rNLRP1 (B), or CARD8 (C) in HEK293T or HEK293T^DPP8/9 DKO^ cells. HEK293T or HEK293T^DPP8/9 DKO^ cells were transfected with indicated plasmids and cell lysates were subjected to IP and IB with indicated antibodies. (**D**) VbP activates both NLRP1 and CARD8 inflammasomes. HEK293T cells were co-transfected with ASC, caspase-1, pro-IL-1β, and NLRP1 or CARD8, followed by VbP (10 μM) treatment for 6 h. The cell lysates were subjected to IB with indicated antibodies at 36 h post-transfection. (**E**) VbP cannot activate the NT-CT autoinhibitory complexes. HEK293T cells were co-transfected with ASC, caspase-1, pro-IL-1β, and indicated NT or CT, followed by VbP (10 μM) treatment for 6 h. The cell lysates were subjected to IB with indicated antibodies at 36 h post-transfection.
(TIF)

S3 Fig Characterization of the NT-CT autoinhibitory complex of mouse NLRP1b. (**A**) Linker3 binds to mNLRP1b^CT^ in HEK293T cells. HEK293T cells were transfected with indicated plasmids and cell lysates were subjected to IP and IB with indicated antibodies. (**B**) Interaction between UPA and mNLRP1b^CT^ in yeast two-hybrid assay. (**C**) Domain architecture of mNLRP1b. (**D**) Interaction between mNLRP1b^CT^ and Linker3 mutants in in yeast two-hybrid assay. (**E**) Quantification of ASC spike microscopy images in GFP–ASC HEK293T cells transfected with wild-type mNLRP1b or mNLRP1b^Δlinker3^ (n = 3). (**F and G**) Activation of mNLRP1b^NT^-mNLRP1b^CT^ complex by LF (F) or 2DG (G). HEK293T cells were co-transfected with ASC, caspase-1, pro-IL-1β, mNLRP1b^NT^, or mNLRP1b^CT^, followed by indicated treatment. The cell lysates were subjected to IB with indicated antibodies at 36 h post-transfection. LT:1 μg/ml for both LF and PA for 12 h. 2DG: 50 mM for 6 h. All data represent three independent experiments. For statistical analysis, two-tailed unpaired Student’s *t*-test in (E).
(TIF)

S4 Fig Characterization of the NT-CT autoinhibitory complex of rat NLRP1. (**A**) Both ZU5 and UPA bind to rNLRP1^CT^ in HEK293T cells. (**B**) ZU5 interacts with UPA in HEK293T cells. (**C**) Domain architecture of rNLRP1^NT^. (**D**) ASC spike quantification in GFP-ASC HEK293T cells transfected with indicated plasmids for 36 h (n = 3). (**E**) LT activates rNLRP1^NT^-rNLRP1^CT^ complex. HEK293T cells were co-transfected with ASC, caspase-1, pro-IL-1β, rNLRP1^NT^, or rNLRP1^CT^, followed by LT treatment. The cell lysates were subjected to IB with indicated antibodies at 36 h post-transfection. LT: 1 μg/ml for both LF and PA for 12 h. (**F**) Schematic diagram for the domain interaction in NT-CT autoinhibitory complexes of NLRP1 paralogs and CARD8. All data represent two independent experiments. For statistical analysis, two-tailed unpaired Student’s *t*-test and one-way ANOVA in (D).
(TIFF)

## References

[1] Broz P, Dixit VM. Inflammasomes: mechanism of assembly, regulation and signalling. Nat Rev Immunol. 2016 Jul;16(7):407–20. doi: 10.1038/nri.2016.58 27291964

[2] Shi J, Zhao Y, Wang Y, Gao W, Ding J, Li P, et al. Inflammatory caspases are innate immune receptors for intracellular LPS. Nature. 2014 Oct 9;514(7521):187–92. doi: 10.1038/nature13683 25119034

[3] Kayagaki N, Warming S, Lamkanfi M, Vande Walle L, Louie S, Dong J, et al. Non-canonical inflammasome activation targets caspase-11. Nature. 2011 Nov;479(7371):117–21. doi: 10.1038/nature10558 22002608

[4] Shi J, Zhao Y, Wang K, Shi X, Wang Y, Huang H, et al. Cleavage of GSDMD by inflammatory caspases determines pyroptotic cell death. Nature. 2015 Oct 29;526(7575):660–5. doi: 10.1038/nature15514 26375003

[5] Kayagaki N, Stowe IB, Lee BL, O’Rourke K, Anderson K, Warming S, et al. Caspase-11 cleaves gasdermin D for non-canonical inflammasome signalling. Nature. 2015 Oct;526(7575):666–71. doi: 10.1038/nature15541 26375259

[6] Shi X, Sun Q, Hou Y, Zeng H, Cao Y, Dong M, et al. Recognition and maturation of IL-18 by caspase-4 noncanonical inflammasome. Nature. 2023 Dec;624(7991):442–50. doi: 10.1038/s41586-023-06742-w 37993714

[7] Taabazuing CY, Griswold AR, Bachovchin DA. The NLRP1 and CARD8 inflammasomes. Immunol Rev. 2020 Sep;297(1):13–25. doi: 10.1111/imr.12884 32558991 PMC7483925

[8] Yang X, Zhou J, Liu C, Qu Y, Wang W, Xiao MZX, et al. KSHV-encoded ORF45 activates human NLRP1 inflammasome. Nat Immunol. 2022 Jun;23(6):916–26. doi: 10.1038/s41590-022-01199-x 35618833

[9] Chui A, Okondo M, Rao S, Gai K, Griswold A, Johnson D, et al. N-terminal degradation activates the NLRP1B inflammasome. Science. 2019 Apr 5;364(6435):82–5. 30872531 10.1126/science.aau1208 PMC6610862

[10] Sandstrom A, Mitchell PS, Goers L, Mu EW, Lesser CF, Vance RE. Functional degradation: a mechanism of NLRP1 inflammasome activation by diverse pathogen enzymes. Science. 2019 Apr 5;364(6435):eaau1330. 30872533 10.1126/science.aau1330 PMC6532986

[11] Xu H, Shi J, Gao H, Liu Y, Yang Z, Shao F, et al. The N-end rule ubiquitin ligase UBR2 mediates NLRP1B inflammasome activation by anthrax lethal toxin. EMBO J. 2019 Jul 1;38(13):e101996. 31268597 10.15252/embj.2019101996 PMC6600268

[12] Robinson KS, Teo DET, Tan KS, Toh GA, Ong HH, Lim CK, et al. Enteroviral 3C protease activates the human NLRP1 inflammasome in airway epithelia. Science. 2020 Dec 4;370(6521):eaay2002. 33093214 10.1126/science.aay2002

[13] Bauernfried S, Scherr MJ, Pichlmair A, Duderstadt KE, Hornung V. Human NLRP1 is a sensor for double-stranded RNA. Science. 2021 Jan 29;371(6528):eabd0811. 33243852 10.1126/science.abd0811

[14] Robinson KS, Toh GA, Rozario P, Chua R, Bauernfried S, Sun Z, et al. ZAKα-driven ribotoxic stress response activates the human NLRP1 inflammasome. Science. 2022 Jul 15;377(6603):328–35. 35857590 10.1126/science.abl6324 PMC7614315

[15] Jenster L-M, Lange K-E, Normann S, vom Hemdt A, Wuerth JD, Schiffelers LDJ, et al. P38 kinases mediate NLRP1 inflammasome activation after ribotoxic stress response and virus infection. J Exp Med. 2023 Jan 2;220(1):e20220837. doi: 10.1084/jem.20220837 36315050 PMC9623368

[16] Zhou JY, Sarkar MK, Okamura K, Harris JE, Gudjonsson JE, Fitzgerald KA. Activation of the NLRP1 inflammasome in human keratinocytes by the dsDNA mimetic poly(dA:dT). Proc Natl Acad Sci U S A. 2023 Jan 31;120(5):e2213777120. 36693106 10.1073/pnas.2213777120 PMC9945980

[17] Wang Q, Gao H, Clark KM, Mugisha CS, Davis K, Tang JP, et al. CARD8 is an inflammasome sensor for HIV-1 protease activity. Science. 2021 Mar 19;371(6535):eabe1707. doi: 10.1126/science.abe1707 33542150 PMC8029496

[18] Wang Q, Clark K, Tiwari R, Raju N, Tharp G, Rogers J, et al. The CARD8 inflammasome dictates HIV/SIV pathogenesis and disease progression. Cell. 2024 Feb 29;187(5):1223–1237.e16. 38428396 10.1016/j.cell.2024.01.048 PMC10919936

[19] Nadkarni R, Chu WC, Lee CQE, Mohamud Y, Yap L, Toh GA, et al. Viral proteases activate the CARD8 inflammasome in the human cardiovascular system. J Exp Med. 2022 Sep 21;219(10):e20212117. doi: 10.1084/jem.20212117 36129453 PMC9499823

[20] Gong Q, Robinson K, Xu C, Huynh PT, Chong KHC, Tan EYJ, et al. Structural basis for distinct inflammasome complex assembly by human NLRP1 and CARD8. Nat Commun. 2021 Jan 8;12(1):188. doi: 10.1038/s41467-020-20319-5 33420028 PMC7794362

[21] Robert Hollingsworth L, David L, Li Y, Griswold AR, Ruan J, Sharif H, et al. Mechanism of filament formation in UPA-promoted CARD8 and NLRP1 inflammasomes. Nat Commun. 2021 Jan 8;12(1):189. doi: 10.1038/s41467-020-20320-y 33420033 PMC7794386

[22] Hollingsworth LR, Sharif H, Griswold AR, Fontana P, Mintseris J, Dagbay KB, et al. DPP9 sequesters the C terminus of NLRP1 to repress inflammasome activation. Nature. 2021 Apr;592(7856):778–83. 33731932 10.1038/s41586-021-03350-4 PMC8299537

[23] Sharif H, Hollingsworth LR, Griswold AR, Hsiao JC, Wang Q, Bachovchin DA, et al. Dipeptidyl peptidase 9 sets a threshold for CARD8 inflammasome formation by sequestering its active C-terminal fragment. Immunity. 2021 Jul 13;54(7):1392–1404.e10. 34019797 10.1016/j.immuni.2021.04.024 PMC8423358

[24] Huang M, Zhang X, Toh GA, Gong Q, Wang J, Han Z, et al. Structural and biochemical mechanisms of NLRP1 inhibition by DPP9. Nature. 2021 Apr;592(7856):773–7. doi: 10.1038/s41586-021-03320-w 33731929 PMC8081665

[25] Boyden ED, Dietrich WF. Nalp1b controls mouse macrophage susceptibility to anthrax lethal toxin. Nat Genet. 2006 Feb;38(2):240–4. 16429160 10.1038/ng1724

[26] Neiman-Zenevich J, Liao K-C, Mogridge J. Distinct regions of NLRP1B are required to respond to anthrax lethal toxin and metabolic inhibition. Infect Immun. 2014 Sep;82(9):3697–703. doi: 10.1128/IAI.02167-14 24935976 PMC4187829

[27] Neiman-Zenevich J, Stuart S, Abdel-Nour M, Girardin SE, Mogridge J. Listeria monocytogenes and *Shigella flexneri* activate the NLRP1B inflammasome. Infect Immun. 2017 Nov;85(11):e00338–17. doi: 10.1128/IAI.00338-17 28808162 PMC5649023

[28] Hellmich KA, Levinsohn JL, Fattah R, Newman ZL, Maier N, Sastalla I, et al. Anthrax lethal factor cleaves mouse nlrp1b in both toxin-sensitive and toxin-resistant macrophages. PLoS ONE. 2012;7(11):e49741. doi: 10.1371/journal.pone.0049741 23152930 PMC3495862

[29] Chavarría-Smith J, Vance RE. Direct proteolytic cleavage of NLRP1B is necessary and sufficient for inflammasome activation by anthrax lethal factor. PLoS Pathog. 2013;9(6):e1003452. doi: 10.1371/journal.ppat.1003452 23818853 PMC3688554

[30] Chavarría-Smith J, Mitchell PS, Ho AM, Daugherty MD, Vance RE. Functional and evolutionary analyses identify proteolysis as a general mechanism for NLRP1 inflammasome activation. PLoS Pathog. 2016 Dec;12(12):e1006052. doi: 10.1371/journal.ppat.1006052 27926929 PMC5142783

