# Supplementary figures and images for: Diverse autoinhibitory mechanisms of FIIND-containing proteins: Insight into regulation of NLRP1 and CARD8 inflammasome
Source: PLoS Pathog. 2025 Jan 24;21(1):e1012877. doi: 10.1371/journal.ppat.1012877 (PMC11760013; doi:10.1371/journal.ppat.1012877)

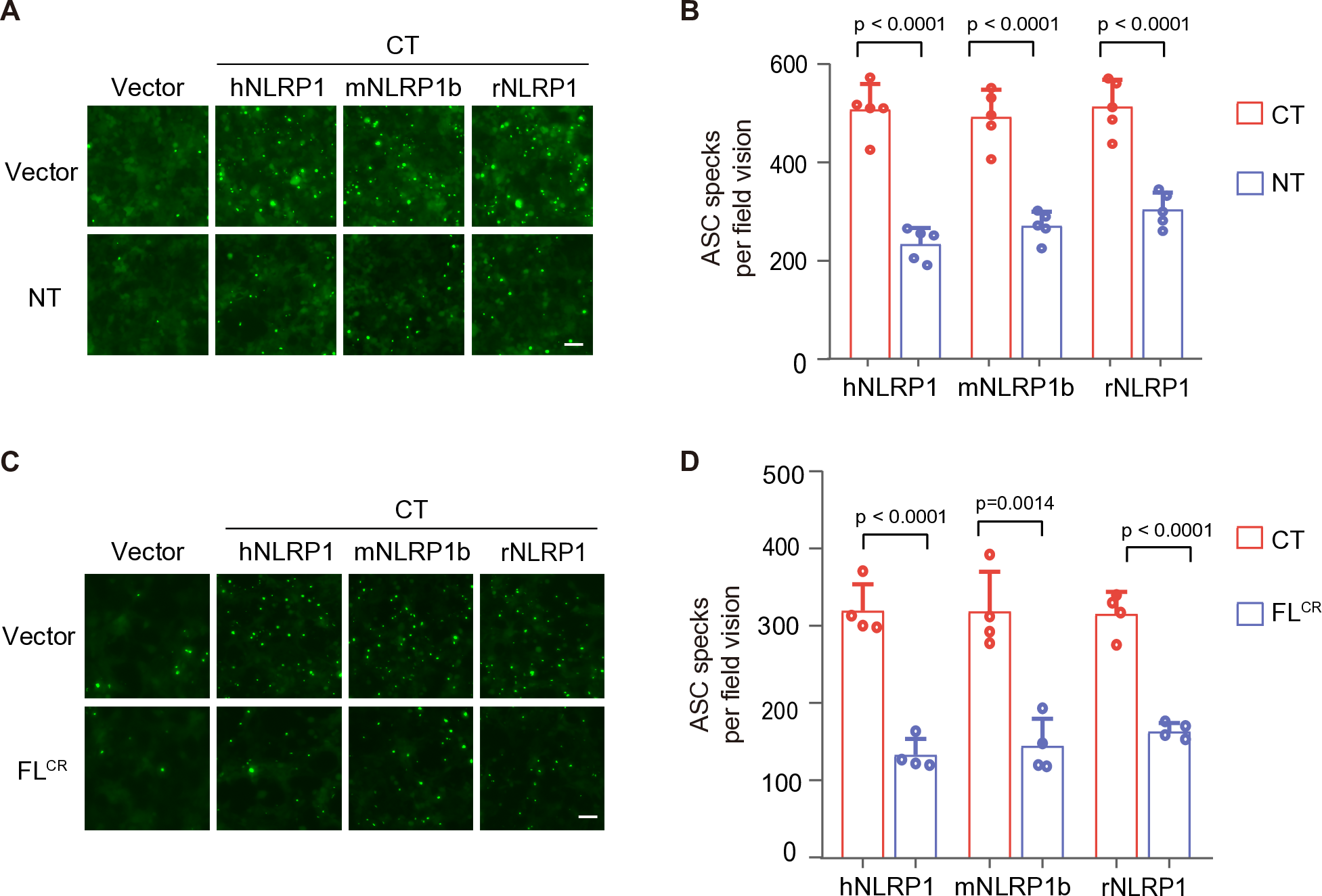

Supplement: S1 Fig — (A and B) ASC spike microscopy images (A) and quantification (B) of GFP–ASC HEK293T cells transfected with expression vectors encoding NLRP1CT and NLRP1NT from different species as indicated for 36 h (n = 5). Scale bar, 50 μm. (C and D) ASC spike microscopy images (C) and quantification (D) of GFP–ASC HEK293T cells transfected with expression vectors encoding NLRP1CT and NLRP1 cleavage-resistant (CR) mutants from different species as indicated for 36 h (n = 5). Scale bar, 50 μm. All data represent three independent experiments. For statistical analysis, two-tailed paired Student’s t-tests in (B, D). (TIF) [file ppat.1012877.s001.tif]

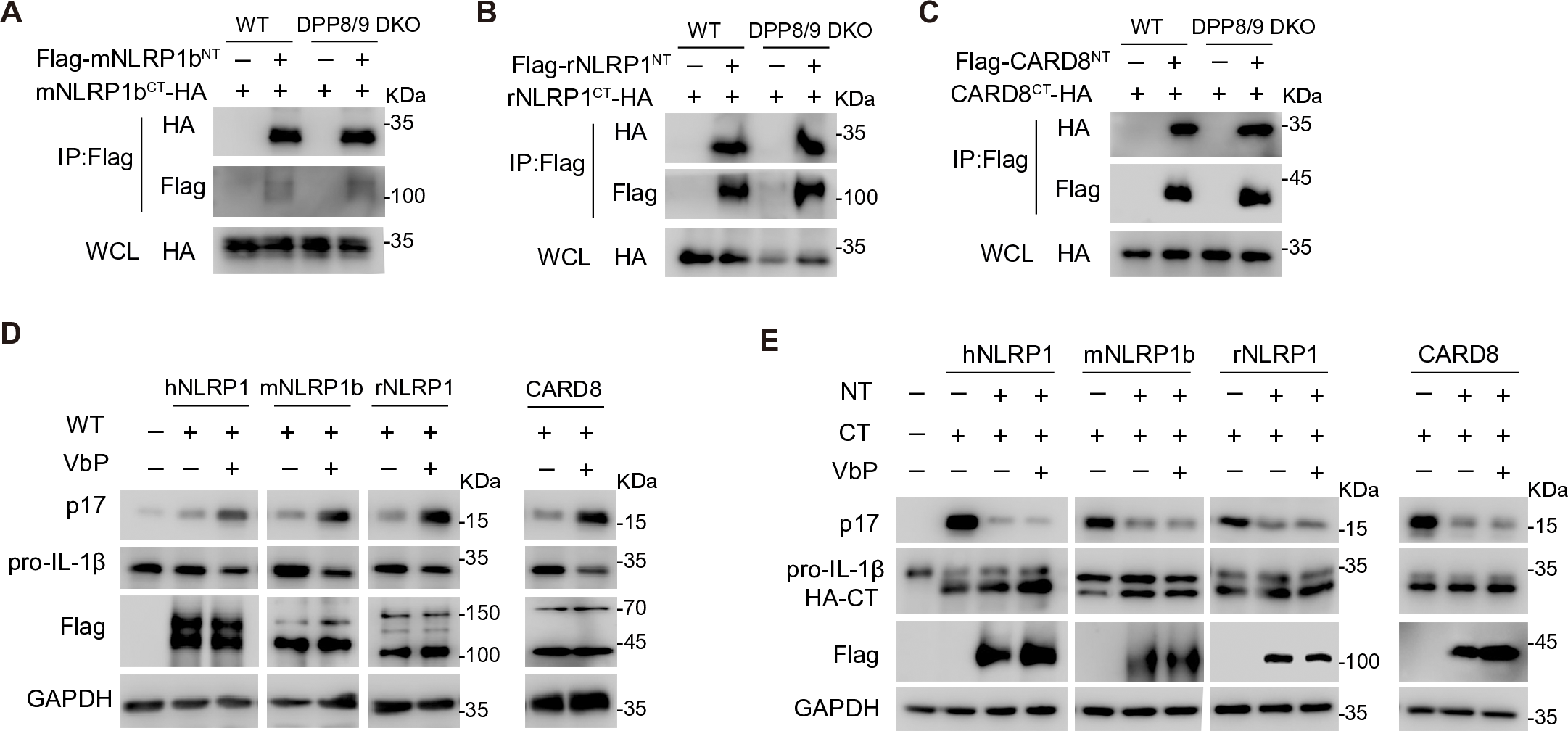

Supplement: S2 Fig — (A–C) Interaction between NT and CT of mNLRP1b (A), rNLRP1 (B), or CARD8 (C) in HEK293T or HEK293TDPP8/9 DKO cells. HEK293T or HEK293TDPP8/9 DKO cells were transfected with indicated plasmids and cell lysates were subjected to IP and IB with indicated antibodies. (D) VbP activates both NLRP1 and CARD8 inflammasomes. HEK293T cells were co-transfected with ASC, caspase-1, pro-IL-1β, and NLRP1 or CARD8, followed by VbP (10 μM) treatment for 6 h. The cell lysates were subjected to IB with indicated antibodies at 36 h post-transfection. (E) VbP cannot activate the NT-CT autoinhibitory complexes. HEK293T cells were co-transfected with ASC, caspase-1, pro-IL-1β, and indicated NT or CT, followed by VbP (10 μM) treatment for 6 h. The cell lysates were subjected to IB with indicated antibodies at 36 h post-transfection. (TIF) [file ppat.1012877.s002.tif]

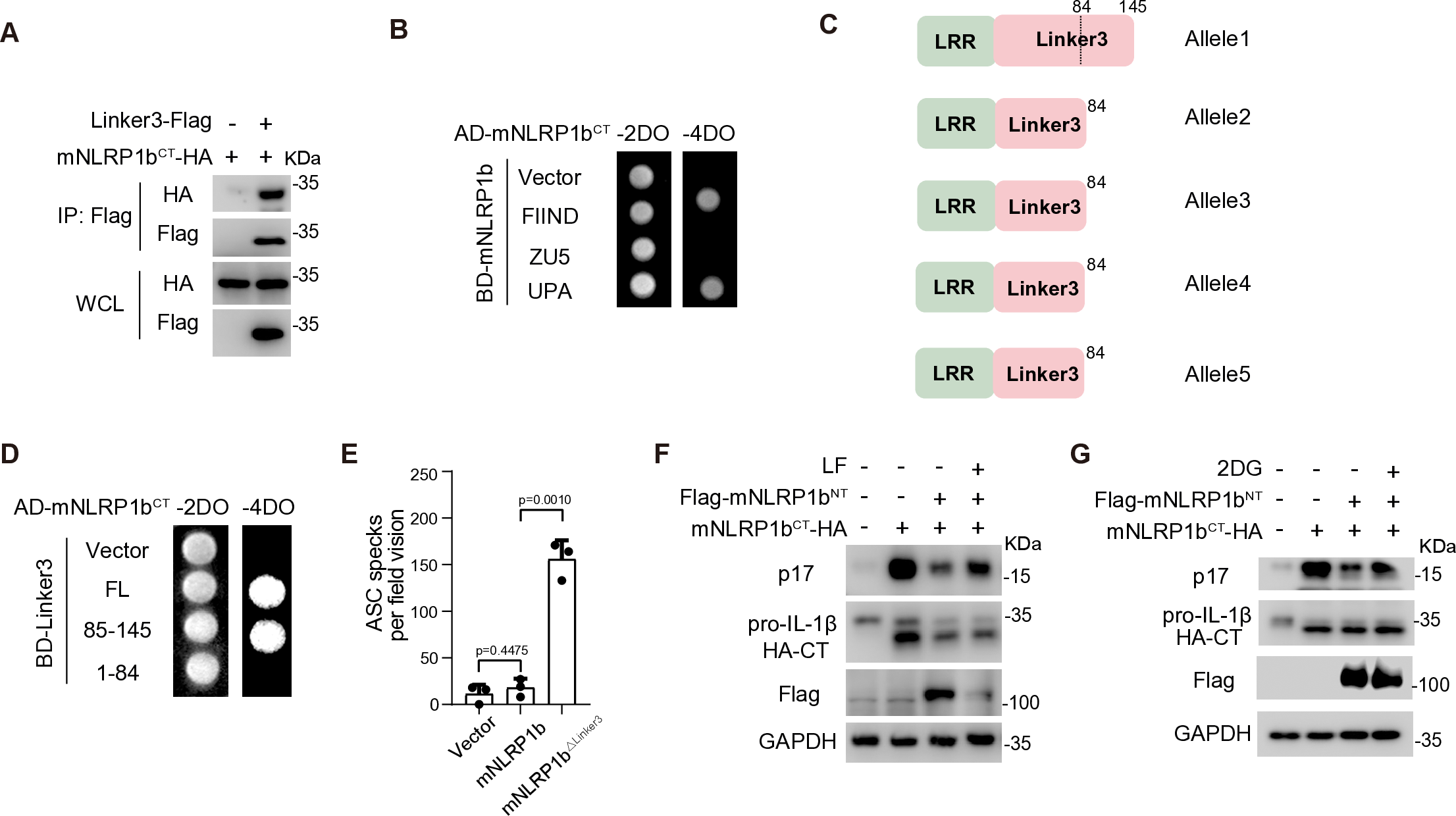

Supplement: S3 Fig — (A) Linker3 binds to mNLRP1bCT in HEK293T cells. HEK293T cells were transfected with indicated plasmids and cell lysates were subjected to IP and IB with indicated antibodies. (B) Interaction between UPA and mNLRP1bCT in yeast two-hybrid assay. (C) Domain architecture of mNLRP1b. (D) Interaction between mNLRP1bCT and Linker3 mutants in in yeast two-hybrid assay. (E) Quantification of ASC spike microscopy images in GFP–ASC HEK293T cells transfected with wild-type mNLRP1b or mNLRP1bΔlinker3 (n = 3). (F and G) Activation of mNLRP1bNT-mNLRP1bCT complex by LF (F) or 2DG (G). HEK293T cells were co-transfected with ASC, caspase-1, pro-IL-1β, mNLRP1bNT, or mNLRP1bCT, followed by indicated treatment. The cell lysates were subjected to IB with indicated antibodies at 36 h post-transfection. LT:1 μg/ml for both LF and PA for 12 h. 2DG: 50 mM for 6 h. All data represent three independent experiments. For statistical analysis, two-tailed unpaired Student’s t-test in (E). (TIF) [file ppat.1012877.s003.tif]

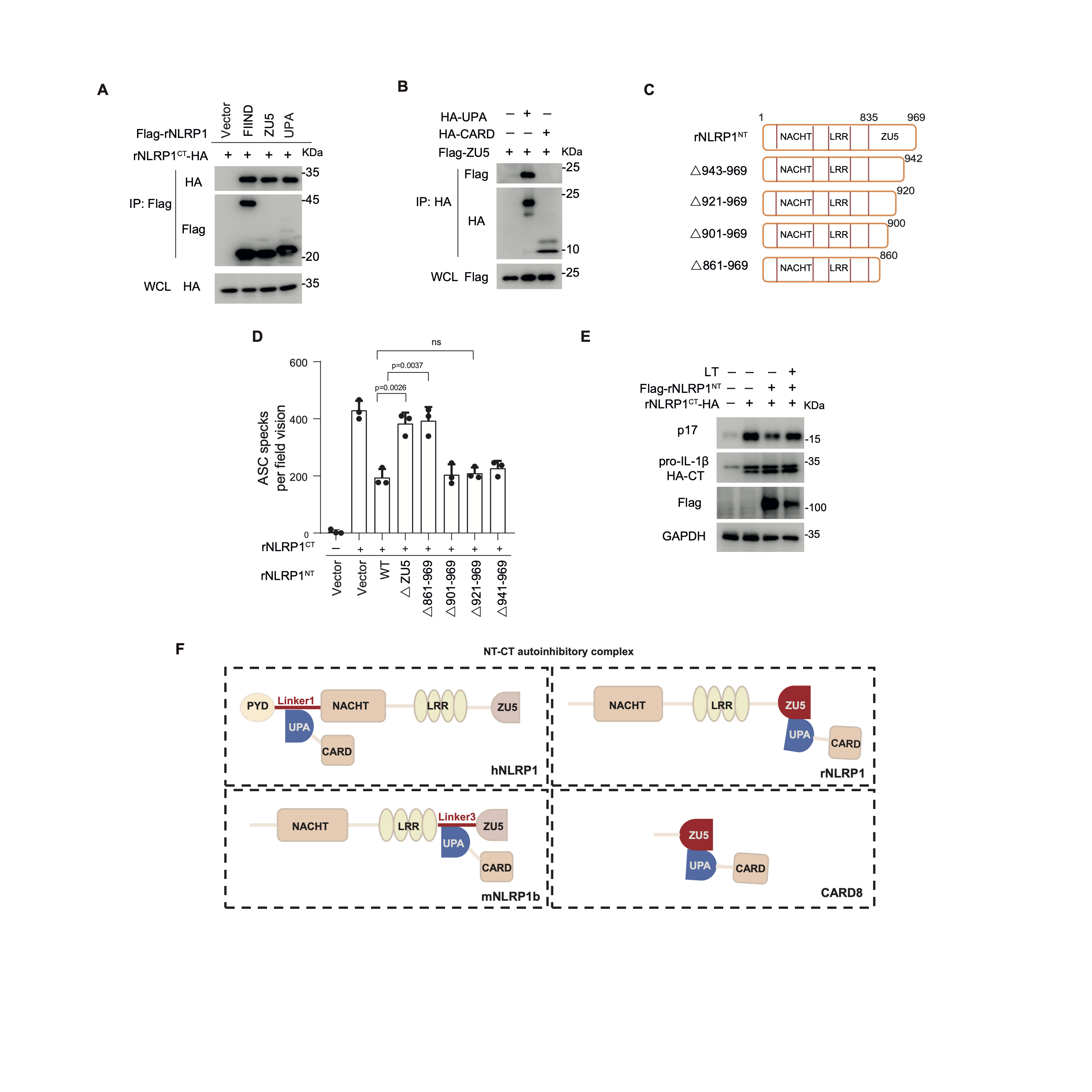

Supplement: S4 Fig — (A) Both ZU5 and UPA bind to rNLRP1CT in HEK293T cells. (B) ZU5 interacts with UPA in HEK293T cells. (C) Domain architecture of rNLRP1NT. (D) ASC spike quantification in GFP-ASC HEK293T cells transfected with indicated plasmids for 36 h (n = 3). (E) LT activates rNLRP1NT-rNLRP1CT complex. HEK293T cells were co-transfected with ASC, caspase-1, pro-IL-1β, rNLRP1NT, or rNLRP1CT, followed by LT treatment. The cell lysates were subjected to IB with indicated antibodies at 36 h post-transfection. LT: 1 μg/ml for both LF and PA for 12 h. (F) Schematic diagram for the domain interaction in NT-CT autoinhibitory complexes of NLRP1 paralogs and CARD8. All data represent two independent experiments. For statistical analysis, two-tailed unpaired Student’s t-test and one-way ANOVA in (D). (TIFF) [file ppat.1012877.s004.tiff]
